# Effect of Femtosecond-Laser-Structured Injection Molding Tool on Mechanical Properties of the Manufactured Product

**DOI:** 10.3390/polym13132187

**Published:** 2021-06-30

**Authors:** Krisztián Kun, Zoltán Weltsch

**Affiliations:** Department of Innovative Vehicles and Materials, GAMF Faculty of Mechanical Engineering and IT, John von Neumann University, 6000 Kecskemét, Hungary; kun.krisztian@gamf.uni-neumann.hu

**Keywords:** surface structures, femtosecond laser, microstructures, mechanical properties, surface modifications

## Abstract

During the injection molding process, the melt travels with a flow due to friction. As the velocity of the layers next to the wall is less than that of those flowing in the middle of the channel, a fountain flow is formed at the melt front. The temperature of the polymer surface decreases from the melt temperature to the contact temperature after contacting the mold surface. Based on all this, a complex shell–core structure is formed in injection-molded products, which can be influenced by the processing parameters and the surface of the tool insert. This paper focuses on investigating the effect of the microstructures replicated from the insert to the polymer product on its mechanical properties. During the research, two microstructured surfaces were created, with different effects on the melt flow formed by the femtosecond laser. These were compared with a ground insert to analyze the effects. For examining the effect of technological variables on the mechanical properties, an experimental design was used. The structure created by the femtosecond laser on the surface of the tool influenced the mechanical properties of the polymer products. Recognizing the effect of microstructures on the melt front and, through this, the change in mechanical properties, a predefined polymer product property can be achieved.

## 1. Introduction

**Figure 1:**
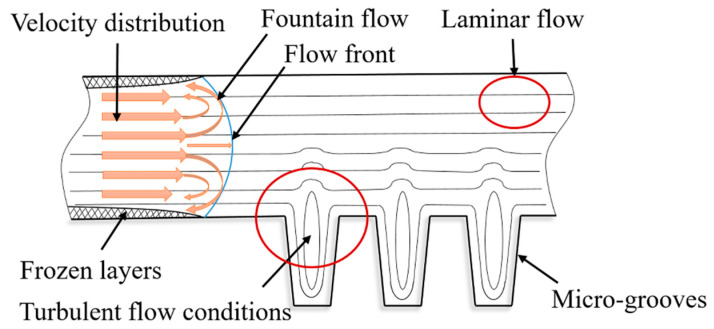
Flow of viscous fluid in cavity with micro-grooves.

During injection molding, the polymer in contact with the cavity surface typically has zero velocity, and at the center, it is maximum. Opposed to this, the shear stress at the cavity wall decreases from its highest value, to near-zero at the center, influenced by the friction conditions. During the injection and holding stage, the mold surface has a lower temperature than the melt temperature. Despite the cooling, the temperature of the mold surface is continuously increased by the hot melt. As shown in Figure 1, a frozen layer is formed close to the cavity walls. Therefore, the microstructures cannot be replicated even when a high holding pressure is used. To minimize this phenomenon, the mold can be heated before the material injection. Thanks to this, the viscosity of the plastic material is maintained at a low level until the microstructures in the cavity are filled. In the case of polymer products with micro-grooves or ribs, the material flow can have different characteristics and heat transfer conditions. The turbulent flow generated by these microstructures increases the pressure of the melt, as well as the shear stress. The pressure increase determines the decrease in melt velocity, and this could cause an increase in the proportion of frozen layers. The flow instability, because of melt velocity and pressure variation, affects the fountain flow and consequently the outer layer on the surface of the finished product [1,2].

There will always be some degree of molecular orientation in the product, which differs in the cross-sections. Zuev et al. investigated the morphology of the multi-layered structures [1]. Due to the pseudoplastic behavior, next to the cavity wall, highly oriented layers developed where the shear rate is higher thanks to the fountain flow, while inside the melt front, the layers were less oriented [2,3,4].

The macromolecules are aligned parallel to the flow direction; otherwise, its front and its end would travel at different rates. In the case of laminar flow, the polymer parts are more relaxed in areas without ribs. However, Porfyrakis proposed that, because of surface microstructures (ribs), the pressure of the material flow increases, as well as the shear stress [5]. The molded material is oriented in the area of the surface structures (ribs). The pressure increase also determines the decrease in flow speed, and this could cause premature solidification. According to Moritomi et al., the changes in flow velocity and cavity pressure affect flow instability [6]. 

In addition to the velocity gradient, there is also a temperature gradient that is quite uniform along the thickness of the flow front. This situation changes quickly soon after the first contact of the polymer with the tool surface due to the effect of heat transfer. Solomon et al. proposed that, if an inappropriate cycle time (especially with regard to the injection speed and cooling time) is determined for the manufacturing technology, then the melt meets the same section of the molding tool wall for a shorter time, thus changing the heat transfer conditions [2]. The cooling time of the product affects the composition of differently oriented layers. Controlling the temperature field and shear stress field can improve the quality of plastic products. Sha et al. observed that, as the microstructure of plastic products is formed under complex forces and temperature fields, whereas the unstable material flow is reflected in the surface topography, the quality of plastic products is mostly controlled by its processing experience [7]. 

The surface structure of the tool changes the filling process and the cooling efficiency. For this reason, it can be observed that the ratio of the layers with different molecular orientations changes. Belina pointed out a phenomenon of polymers that the mechanical properties can also change as a result of molecular changes [3]. However, to understand the effect of surface structures on orientation, we must first understand the filling process. Based on these, the mechanical properties of plastic products depend not only on the properties of the materials themselves but also on the microstructure. The replication quality of molded parts or even their mechanical properties can be improved by optimizing process parameters and controlling material flow. Therefore, an optimization approach has been used to improve the mechanical properties of the molded part made by polypropylene (PP) [4,8,9,10,11,12].

The aim of the research was to investigate the effect of the replicated microstructures manufactured with optimal injection molding parameters on the polymer products’ mechanical properties. In order to be able to investigate the effect of the two different microstructures and to compare the polymer products to standard specimens, an insert with the ground surface was also machined [13,14,15,16,17,18].

## 2. Materials and Methods

Examining the properties of plastics is important for the choice of their areas of application. The molding tool’s forming surface structure has a great influence on the filling process and, thus, on the molecular orientation and on the cooling efficiency. The researchers hypothesized that this effect could also affect the property characteristics of the material, thus changing the mechanical properties of the injection-molded product as well. In the course of the research, three-point bending and Charpy impact bending tests were performed on products with different surface structures.

A special injection mold was designed and manufactured, which included two symmetrical cavities. The products of the molding tool were two bending specimens. The aim was to create two surface structures on the A plate’s insert, which would affect the melt flow differently during injection molding. In the present research, our goal was to create a uniform surface that contains micro-grooves with close to the same depth. The texturing of the surface was formed by a femtosecond laser.

### 2.1. Structuring of the Cavity Using a Femtosecond Laser

A common feature of laser beam material processing technologies is that the material separation forms a constant and unified surface. The small beam size of a femtosecond laser also makes it possible to process details of microscopic size and generate short pulses that do not make thermal marks on the machined part (heat-affected zone). During the cold ablation process, the removed material changes from a solid to a gaseous state without an intermediate state.

**Figure 2:**
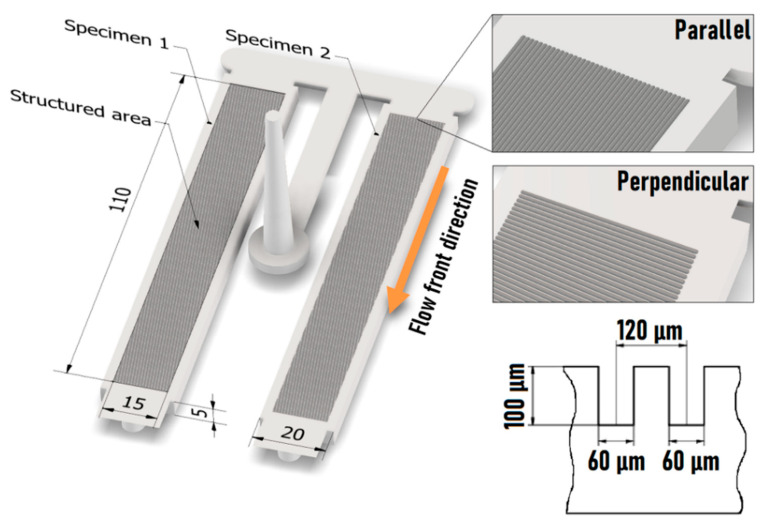
The molded products with microstructured surfaces and the significant dimensions.

Perpendicular and parallel groove geometries with a depth of 100 µm were formed (Figure 2). These were machined by a Monaco 1035-80-40 industrial femtosecond laser. The laser beam was made using a LINOS F-Theta-Ronar scanner optics with a f = 254 mm focal-length lens. The goal was to apply a manufacturing technology that can achieve a uniform microstructure (Figure 2) on the injection mold insert. The laser process parameters were set to make the geometries as uniform as possible and the heat zone as small as possible. Particular attention was paid to avoiding technology-specific errors such as plasma formation, possible melt residues, and oxidation [19,20].

According to several research studies, the result of femtosecond laser machining is influenced by several factors [4,17,21,22,23]. Prominent surface quality can be achieved on thin (>0.5 mm) materials. However, for thicker materials, it is much more difficult to achieve a similar result. As far as the parameters are concerned, there is no consensus among researchers: different pulse widths, frequencies, and power values effect various results [4,7,10,21,22,24]. By using a femtosecond laser, controlled material removal processing can be expected with a quality, uniform surface, where burr formation is minimal. The cavity inserts had to be surface-treated under argon gas during machining.

The laser machining technology was investigated in an additional experimental design on specimens designed for this purpose, but not discussed in this publication. The applied technology managed to achieve the geometry that best-suited the goal. The beam source of the laser beam was in TEM00 mode; therefore, the intensity distribution of the beam followed a Gaussian distribution. In our case, the intensity was highest in the middle of the beam, gradually decreasing outward. In order to create the planned depth, the structures were made by multiple scans (repeated 30 times in succession). During dynamic focus control, the machine first focused on the top of the surface, resulting in a 60 μm wide channel. After the focus shift, the channel width (60 μm) would still have been made constant, but due to the divergence of the beam, the ablation limit was also reached in the positive direction from the focus position, which further widened the channel. Scattered radiation within the channel removed additional material from layers closer to the surface due to multiple scans.

During the machining, the maximum pulse energy was 80 µJ and the pulse frequency was 188 kHz. The pulse width was 277 fs, and the laser operated at 25% power compared to the average power of 60 W (Table 1). The technology is able to generate very short pulses of a few hundred femtoseconds in length, so even though the average power remains low, the peak power can reach up to 280 MW.

An attempt was made to change two technological values for the appropriate machining settings. These were based on previous research [25]: the scan velocity and the frequency range. The pulse-to-pulse overlap has a significant impact on both quality and productivity. At a lower frequency range, the scan velocity is slow, meaning a larger pulse-to-pulse overlap, resulting in an enhanced ablation rate. A higher pulse overlap increases the thermal effects (oxide layers, molten parts, etc.), thereby degrading the accuracy of the desired surface. In the present publication, the parameters giving the best results were used, which were determined on the basis of the preliminary experiment.

### 2.2. Analysis of the Microstructure Formed on the Cavity’s Surface

**Figure 3:**
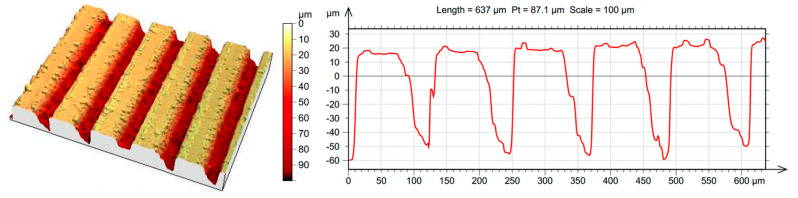
Microscopic image of the cavity’s surface with the designed microstructure dimensions.

After forming micro-grooves, parallel and perpendicular to the melt flow on the surface of the mold insert, these were examined using a 3D optical surface metrology system, Leica DCM8 (Figure 3). The main question was whether the planned depth of 100 µm was reached.

It can be clearly seen in the figure that the average structure depth was 87.1 µm, which approximated the planned depth of 100 µm. The ribs formed on the polymer product by the insert increased the volume of the entire element by less than 0.8% (based on the CAD model). This only happened if the cavity was completely filled by the melt [12,17,18].

### 2.3. The Polymer Material Used for Injection Molding

The specification of the mold tool’s geometric boundary conditions was followed by the choice of polymer material. It was obvious to use a material that typically occurs in an area of industry where microstructures can be encountered. Due to increasing customer demands, engineers (especially in the automotive industries) create microstructures on the surface of polymer products, close to the appearance of natural materials. 

The choice of raw material was made of polypropylene (PP), which is a thermoplastic crystal-line polymer. In the past decade, the rheology, phase morphology, and thermal- and mechanical properties of PP specimens were studied. This material is suitable for research because of its high flow ability, excellent processing stability, and the field of use. Typical applications are transparent cases, ribbed lids, etc. Its physical properties are shown in the Table 2 [26].

### 2.4. Injection Molding Conditions and Preliminary Experimental Study to Examine the Replication Quality

Several process parameters could affect the quality of the molded part and its surface replication. The main factors investigated by researchers were melt and mold temperature, and injection rate and pressure. As the applied experimental injection molding tool was not equipped with temperature sensors, the effects of melt temperature and mold temperature were not investigated. Many researchers, including Su et al. and Sha et al., came to the conclusion that process factors could improve the replication of microstructures [7,27]. Our previous experimental study was conducted to analyze the effect of different processing parameters on the ribs’ replications. The Taguchi experimental design is a traceable and efficient method, which allows us to find the optimal combination of process parameters. Using the appropriate processing technology, a quality product with the most fulfilled depth of microstructures can be produced. The mean values of the manufacturing technologies were defined based on the performance of the injection molding machine and the properties of the raw material. The settings are summarized in Table 3. During injection molding, a robot was used to remove the part after ejection [20,28,29].

**Figure 4:**
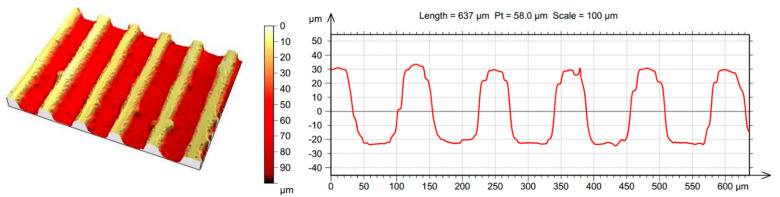
Microscopic view: Test specimen, manufactured with high injection rate (104 cm^3^/s), parallel ribs.

A total of 9 different experiments were performed and six specimens were injection-molded for each experiment to ensure that the product-specific setting was correct (Figure 4). The preliminary study found that the injection speed had the most significant effect on the filling. As the speed increased, the filling of structured surfaces also increased. It can also be observed that the filling of parallel ribs was significantly better. Increasing the packing pressure to the mean value (355 bar) further improved the replication, but raising it no longer yielded significant results (Table 4). The difference between the cooling times used did not cause a visible difference in the filling. Gim et al. also concluded the fact that the higher injection speed could add more melt into the microstructures before the maximum cavity pressure was reached [9]. This was due to an increased injection rate, leading to a decrease in melt viscosity that improves the melt flow in microstructures. The injection rate in the interaction with the packing pressure had a positive effect on the replication quality, because the melt flowed further in the micro-cavities at the moment when the main cavity was already completely filled. The lower process parameters led to a freeze of the outer layer of the melt; however, the flow front continued to advance. The continuous build-up of the pressure drove the melt flow further in the surface, and as a result, the newly added melt and the frozen layer formed the microstructures. However, this phenomenon resulted in less filled structures than molding with higher process parameters [30,31].

The parallel and perpendicular microstructures were separated, taking a 3-3 measurement area at the beginning, middle, and end of the track on each surface.

Comparing Figure 3 and Figure 4 shows the quality of replication. As seen in Table 4, the quality was strongly dependent on the injection rate; furthermore, it can be said that at certain processing parameters, the height of the formed ribs reached 60% of the designed microstructure. However, in terms of the total volume of the microstructure, the filling was even higher considering that the structure narrowed downward.

### 2.5. Three-Point Bending Test

During the three-point bending test, the rectangular-shaped injection-molded specimens were loaded as a two-support beam with a central load. The deformation of the specimens can be inferred from the magnitude of the force acting and the degree of deflection. In the evaluation, the modulus of elasticity (stiffness of the material) and the flexural strength can be determined by knowing the maximum bending moment. An accredited Instron 3366 universal mechanical material testing machine was used for bending. The direction of the melt flow was marked on the specimens and placed on the machine with the same side and surface structure in each case. The distance between the supports was set to 64 mm. The correct choice of crosshead speed required special attention, as at higher speeds, the material can behave more rigidly, and its strength and modulus can also give higher values. In our case, the maximum bending value was chosen to be 15 mm, where the pieces have not yet been broken. During the bending test, 6 specimens were used per experiment.

Measuring parameters during three-point bending test:

- Standard: ISO 178
- Measuring range: 10 kN
- Sampling density: 100 ms
- Crosshead speed: 5 mm/min
- Temperature: 22 ± 1 °C and 45 ± 5% relative humidity

### 2.6. Impact Bending Test

The impact bending test is basically suitable for the dynamic testing of materials and their resistance to brittle fracture. The impactor used was an Instron Ceast Impactor Type II device. In order for a uniform, comparable fracture, it is necessary to notch the specimens. The depth of the “V” shape was 1 mm on each specimen. The pieces were always placed on the support brackets of the machine, with the structured or the ground surface facing downward, and care was taken to place the incision on the opposite side to the impact.

Measuring parameters during impact bending test:

- Standard: ISO 179
- Test hammer size: 5 J impact energy
- Support distance: 60 mm
- Notch type: V-shape, 1 mm depth
- Temperature: 22 ± 1 °C and 45 ± 5% relative humidity

### 2.7. Design of Experiment

Following Taguchi’s philosophy, a strategy was developed at which the variables do not interact with each other. Similar to the preliminary experimental design used in the replication study. Based on previous experience, three factors had to considered, namely injection rate (A), packing pressure (B), and cooling time (C). In order to create three variables, deviations of −30% and + 30% from the mean value were set. The experimental design is shown in Table 5. A total of 3 factors were examined at 3 different values, thus generating 33, i.e., 27, different settings, forming a complex experimental design. For each of the 27 types of parameter combinations, 12-12 specimens were molded with both the structured and ground inserts in order to examine the surface-specific effect.

Polypropylene is a semi-crystalline polymer, so the melting of the crystalline phase requires additional heat during the process and, when cooled, the crystallization needs a higher heat dissipation compared to the amorphous phase. During the process, the density also changes: the crystalline phase has a higher density than the melt or the amorphous phase. Due to this phenomenon, the shrinkage of the semi-crystalline polymers is significantly greater, in the general case, 1.5–2.5%. In addition, a further approx. 1% post-shrinkage may occur, so the mechanical measurements were only started 3 days after production [18,29].

## 3. Results and Discussion

The aim of the study was to explore the differences between the two different surface structures and to compare them with products made with a ground insert. Mechanical tests were performed on specimens manufactured according to the experimental design.

### 3.1. Evaluation of the Experimental Design—Three-Point Bending Test

**Figure 5:**
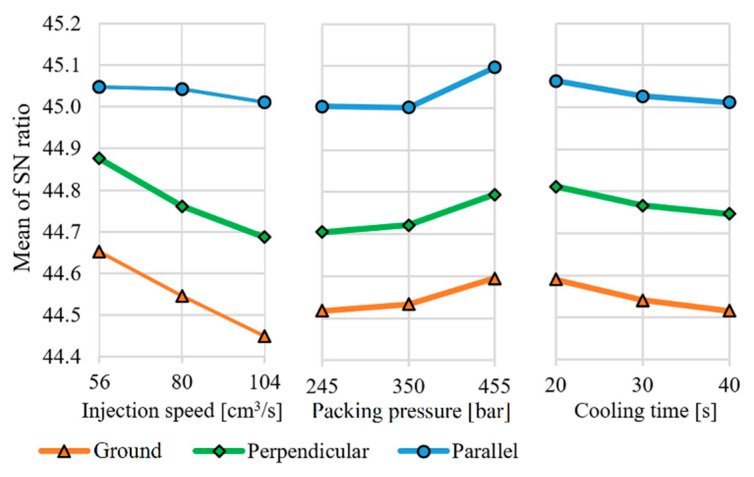
Main effects plot for SN ratios. Three-point bending.

A main effects plot (Figure 5) was performed in the software to determine how technological parameters affect each mechanical property (Table 6). The purpose of the evaluation was to determine which factors affect the mechanical properties and which affect their variance. The Taguchi experimental design calculates with signal-to-noise ratios (SN) closely related to robustness. The signal-to-noise ratio filters out the increase or decrease from the standard deviation, caused by the mean. The formula used for evaluation was the larger is better ratio (1) [32].

(1) $${\mathrm{SN}}_{L}=-10\log\left(\Sigma_{i}\left(\frac{1}{y_{i}^{2}}\right)/n\right)$$

- y is the responses for the given factor level combination;
- n is the number of responses.

Evaluating the experimental design using the measurement results, the following facts can be established: Regarding the technological process parameters, the injection rate has a significant effect in the case of specimens with replicated ground surface and with parallel and perpendicular microstructures. Both the packing pressure and the cooling time had less effect on the measured values of bending force. As the injection rate increased, the amount of bending force (and thus the bending stress) decreased. This can be explained by the higher level of orientation within the product. The increase in cooling time had a similar but smaller effect on the values. In contrast, increasing the packing pressure from 350 to 450 bar increased the bending force, probably due to the better filling of the product. The increase in product weight, in addition to the previous replication experiment, may confirm this fact [18]; however, weight measurement was not investigated in the research. Comparing the products by surface structures, it can be seen that the specimens replicated with the ground insert showed a lower bending force, while the specimens replicated with the laser-structured insert showed a higher bending force. The polymer specimens with parallel microstructures were notable, where the bending force was also less influenced by the process parameters. Compared to the other two structures, a different rank for main effects can be seen, which can be explained by the fact that the microstructure itself had a greater effect on injection molding process than the applied technological parameters did.

### 3.2. Evaluation of the Experimental Design—Impact Bending Test

In the case of the impact bending test, even before the evaluation of the experimental design, it could be observed from the measured data that it was mainly the processing parameters that determined the results. Therefore, the experimental design evaluation interaction was also examined. This makes the plot for mean graphs more informative. 

**Figure 6:**
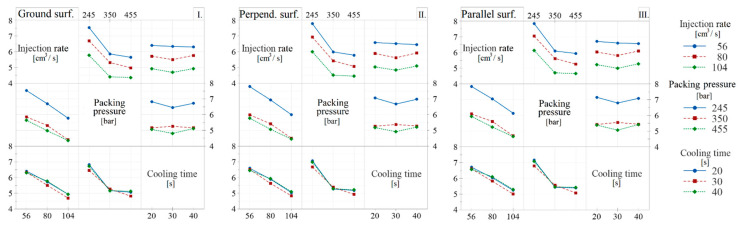
Interaction plot for mean graphs to investigate the effects of the technological parameters on impact strength. The left panel shows the ground (I.), the center shows the perpendicular (II.), and the right (III.) shows the specimens with parallel surface structures.

In terms of the technological process parameters, the packing pressure had the greatest effect on the impact strength in the case of the examined structures. The injection rate had a slightly lower impact on the values than the packing pressure, while the increase in the cooling time did not show a significant change in the impact strength values (Table 7). The interactions of the parameters with each other were also examined (Figure 6). As the impact strength values were mainly influenced by the technological process parameters, the analysis of the interactions was justified in this case. The effect of surface structures on impact strength did not provide a clear response, although a slight increase was observed due to the more favorable molecular orientation for parallel-structured products.

### 3.3. The Results of the Three-Point Bending Test

Thanks to the analysis of the main effect of the experimental design, the bending diagrams can be compared. The main effect analysis pointed to the fact that the injection molding rate had the greatest effect on the bending force. The highest bending force values were produced by the specimens at high packing pressure, while the lowest effect was produced by changing the cooling time [10].

**Figure 7:**
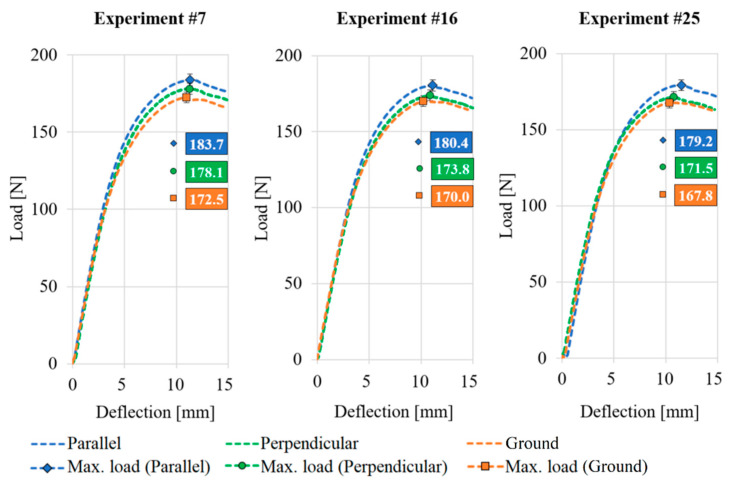
Bending diagrams of specimens made by perpendicular and parallel surface-structured and ground mold inserts.

Each diagram shows three curves, illustrating the difference between the three surface structures. Therefore, the experimental numbers with the highest packing pressure and the lowest cooling time were selected from the experimental design. Three groups of diagrams were distinguished according to the main effect (injection rate), while keeping the other two at a constant value favorable for the bending force mentioned before (Table 8). The three selected experiments can be seen in Figure 7.

If the specimen does not break even at a deflection of 10% of the support distance, the flexural strength is used instead of the tensile strength to characterize it. The bending stress for a rectangular sample under a load in a three-point bending setup is given by the formula below (2):

(2) $$\sigma_{h}=\frac{3\mathrm{FL}}{2{\mathrm{bh}}^{2}}\;\left[\mathrm{MPa}\right]$$

- F is the load (force) at the fracture point (N) and L is the length of the support span;
- b is width and h is the thickness.

The bending stress values determined by Equation (2) and the percentage differences between them are shown in Table 9. Compared to the product with the ground surface, the specimens with perpendicular ribs had a higher bending stress in all examined cases. Nevertheless, the positive effect (>3%) of the perpendicular microstructure could not be clearly expressed. In contrast, the increase in bending stress for products with parallel ribs (>6%) was clearly due to the beneficial effect of the microstructure. The microstructure was created on the product as a positive material (0.5% additional material—knowing the quality of the replication—compared to the specimens with ground surface).

The possible reason is that the perpendicular microstructures provides resistance to the melt flow, slowing the flow rate, thus affecting the quality of the filling and the mechanical properties. The slowdown in the melt front was demonstrated by researchers in an earlier publication [33]. Examining the three experiments side by side with the increase in the value of the injection velocity, a more elastic behavior of the specimens during bending can be observed. It can be explained by the fact that the melt at a temperature of 200 °C enters the cavity at a high injection velocity; thus, it comes into contact with its inner wall for less time, thus deteriorating the heat transfer conditions of the process.

### 3.4. The Results of the Impact Bending Test

**Figure 8:**
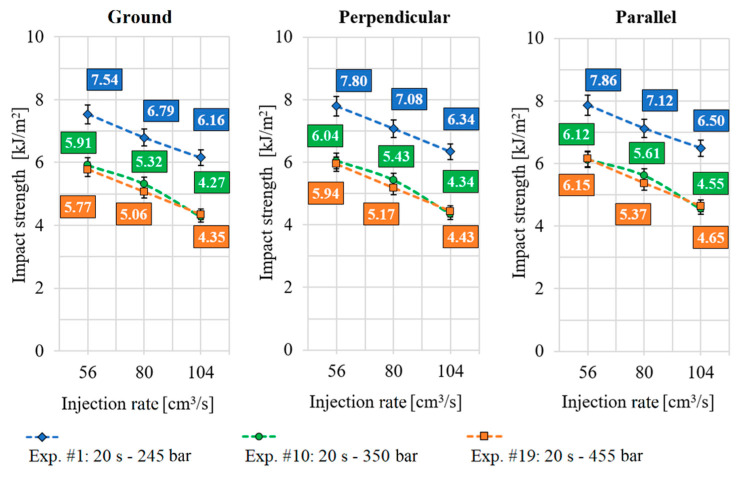
Impact bending test results of specimens made with ground-, perpendicular- and parallel surface-structured inserts.

The impact test diagrams were grouped according to the two most significant factors from the experimental design (injection rate and packing pressure). The specimens with ground, perpendicular, and parallel surface structures were plotted in separate diagrams. The diagrams contain 3-3 curves and each curve represents three experimental results, as shown in Figure 8.

It can be stated from the diagrams that the impact strength values of the products with microstructures were higher in the case of parallel and perpendicular ribs as in the case of the specimens manufactured by the ground surface insert. The percentage differences between the measured values are shown in Table 10.

From the comparison of the measurement results, it can be concluded that, in contrast to the bending stress, the technological process parameters of the injection molding had a significantly greater effect on the impact strength than the presence of the microstructures did.

### 3.5. Regression Analysis

The relationship between input and output parameters of the injection molding process was also examined by the analysis of variance (ANOVA) technique. The regression equation was used to describe the relationship between the response and the terms in the model [32]. The regression equation was used with coded units, where a low factor level was −1 and a high factor level was +1. The response surface contained curvature, so a polynomial model was used, complemented with the interactions. Based on these, a full quadratic model was used for the goodness of the result. These model summaries are collected in Table 11.

The second-order model (3):

(3) $$Y=\beta_{0}+\overset{k}{\underset{i=1}{\sum }}\beta_{i}x_{i}+\overset{k}{\underset{i=1}{\sum }}\beta_{\mathrm{ii}}x_{i}{}^{2}+\sum \underset{i<j}{\sum }\beta_{\mathrm{ij}}x_{i}x_{j}+\varepsilon$$

The equation was solved based on all three surface structures and two different mechanical test results. The correlation coefficients R^2^ that fitted the models indicated the model’s precisions. The adjusted determinations, due to the interactions (different surface structures and processing parameters), resulted in different precisions. 

The applied α = 0.05 was better for the precision and reliability of the experiments. It can be stated that the *p* values of first three factors of the six different regressions (two ground surface, two perpendicular, and two parallel) were lower than that. These significant process parameters are ranked in the last row of Table 11. A lower value of R^2^ was observed at the three-point bending. This was due to the fact that the regression analysis focuses on the effects and interactions of the process parameters; however, the mechanical properties of the products were also influenced by the parallel microstructure itself.

**Figure 9:**
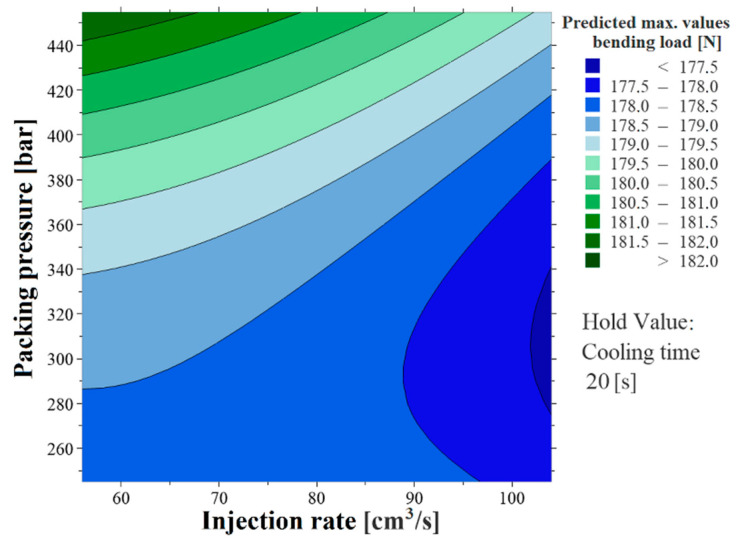
Three-point bending test response surface regression—contour plot of the result of specimens with parallel microstructures.

**Figure 10:**
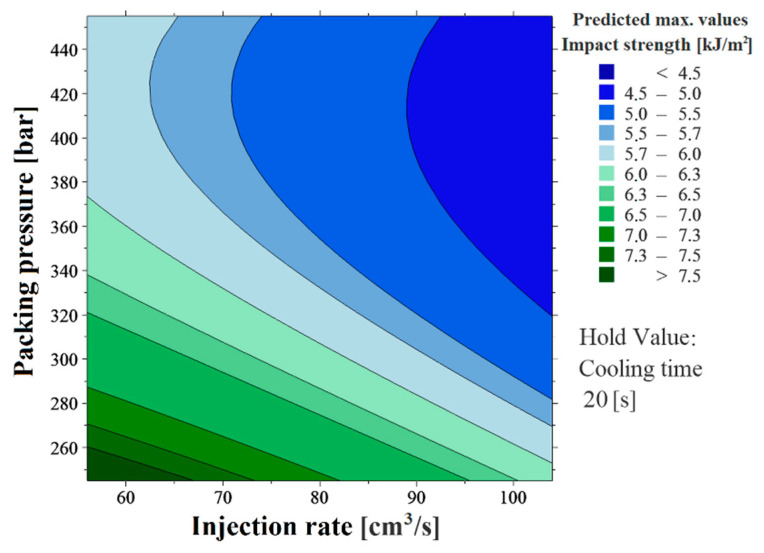
Impact bending test response surface regression—contour plot of the result of specimens with parallel microstructures.

The regression equation is an algebraic representation of the response contours. With a large measured dataset and a high percentage of model results, it can be stated that the generated contour plots can predict the specimens’ mechanical properties with good accuracy. This presupposes industrial applicability. As a result of the research, two contour plots of the surface structure giving the products the highest mechanical properties (specimens with parallel ribs) were formed for the two examined methods. Using the contour plots in Figure 9 and Figure 10, the maximum values of the bending load (Figure 9) and the impact strength (Figure 10) of the plastic specimens can be predicted from the combination of the two most influential factors, taking into consideration the interactions.

## 4. Summary and Conclusions

In the case of polymer products with ribs, the melt flow can have different characteristics and heat transfer conditions. The turbulent flow generated by these microstructures increases the pressure of the melt, as well as the shear stress, and at the same time, decrease the melt velocity. The flow instability affects the fountain flow and, consequently, the outer layer on the surface of the polymer product. For this reason, it can be observed that the ratio of the layers with different molecular orientations changes, so, as a result, the mechanical properties can also change. Based on these, the mechanical properties of plastic products depend not only on the properties of the materials themselves, but also on the surface microstructures. 

The research with its measurement results pointed out that the microstructures created with the femtosecond laser on the surface of the injection molding tool insert affected the mechanical properties of the polymer products. The measurements provided reliable results thanks to the large number of test pieces. Compared to the plastic parts with the ground surface, the specimens with microstructures changed the mechanical properties in both testing methods. In the case where the microstructure has a favorable orientation to the melt flow, its presence may increase the mechanical properties of the products. However, this phenomenon interacted with the process parameters, so the rate of effects varied in different types of mechanical testing methods.

Evaluating the experimental design of the bending test results, the following facts can be established: Regarding the technological process parameters, the injection rate had a significant effect on the measured values, despite the packing pressure and the cooling time. As the injection rate increased, the amount of bending force (and thus the bending stress) decreased. This can be explained by the higher level of orientation within the product. The increase in cooling time had a similar but smaller effect on the values. In contrast, increasing the packing pressure from 350 to 450 bar increased the bending force, thanks to the better filling of the product. Comparing the products by surface structures, the polymer specimens with parallel microstructures were notable, where the bending force was also less influenced by the process parameters. The improvement in mechanical properties in the examined experiments, thanks to process parameters and microstructures, was more than doubled in favor of the geometry.

From the comparison of the measurement results, it can be concluded that, in contrast to the bending stress, the technological process parameters of the injection molding had a significantly greater effect on the impact strength than the presence of the microstructures did. The effect of surface structures on impact strength did not provide a clear response, although a slight increase was observed due to the more favorable molecular orientation for parallel-structured products.

At the end of the research, the results were used to generate a response surface from the measurement data, predicting the possibility of industrial applicability.

## Figures and Tables

**Table 1:** The used technical parameters of the Monaco 1035-80-40 femtosecond laser.

Applied Technical Specification	
Wavelength [nm]	1035+/−5
Applied power [W]	20
Energy [μJ]	80 (at 500 kHz)
Frequency range [kHz]	188
Pulse width [fs]	270
Modus	TEM00
Scanning velocity [m/s]	5

**Table 2:** The properties of the examined polymer.

Properties of TATREN RM 85 82 Clear		Unit
Material type and degree of crystallinity:		
PP (Crystalline) Random copolymer		
Recommended processing temperature:		
Melt temperature	200	°C
pvT properties:		
MFI (Melt flow index)	85	g/10 min
Mechanical properties:		
Tensile stress at yield	30	MPa
Tensile strain at yield	12	%
Flexural modulus	1250	MPa

**Table 3:** Parameters of injection-molding process.

Dose	Filling Parameters	Compression
Peripheral speed	Switching pressure	Switching point
200 mm/s	350 bar	14 cm^3^
Ram pressure	Injection rate (A)	Packing pressure (B)
50 MPa	80 cm^3^/s ±30%	350 bar ±30%
Injected dose	Pressure	Cooling time (C)
40 cm^3^	2000 bar	30 s ±30%

**Table 4:** Mean heights of microstructures on polymer products.

Injection Rate [cm^3^/s]	Perpendicular Structures [µm]	Parallel Structures [µm]
56 (−30%)	21.8	37.8
80 (mean)	30.8	45.7
104 (+30%)	42.4	54.9

**Table 5:** Experimental design with 27 experiments.

Exp. #	Injection Rate [cm^3^/s] (A)	Packing Pressure [bar] (B)	Cooling Time [s] (C)
1.	56	245	20
2.	56	245	30
3.	56	245	40
4.	56	350	20
5.	56	350	30
6.	56	350	40
7.	56	455	20
8.	56	455	30
9.	56	455	40
10.	80	245	20
11.	80	245	30
12.	80	245	40
13.	80	350	20
14.	80	350	30
15.	80	350	40
16.	80	455	20
17.	80	455	30
18.	80	455	40
19.	104	245	20
20.	104	245	30
21.	104	245	40
22.	104	350	20
23.	104	350	30
24.	104	350	40
25.	104	455	20
26.	104	455	30
27.	104	455	40

**Table 6:** Response table of means—three-point bending test, with different surface-structured specimens.

Ground Surface				Perpendicular Structures				Parallel Structures			
Level	Injection Rate [cm^3^/s]	Packing- Pressure [bar]	Cooling Time [s]	Level	Injection Rate [cm^3^/s]	Packing- Pressure [bar]	Cooling Time [s]	Level	Injection Rate [cm^3^/s]	Packing- Pressure [bar]	Cooling Time [s]
1	170.8	168.2	169.7	1	175.3	171.9	174.0	1	178.8	177.9	179.1
2	168.8	168.5	168.7	2	173.0	172.2	173.1	2	178.7	177.9	178.4
3	166.9	169.7	168.2	3	171.5	173.3	172.7	3	178.1	179.8	178.1
Delta	3.9	1.5	1.5	Delta	3.8	1.4	1.3	Delta	0.7	1.9	1.0
Rank	1	2	3	Rank	1	2	3	Rank	3	1	2

**Table 7:** Response table of means—impact bending tests with different surface-structured specimens.

Ground				Perpendicular Structures				Parallel Structures			
Level	Injection Rate [cm^3^/s]	Packing- Pressure [bar]	Cooling Time [s]	Level	Injection Rate [cm^3^/s]	Packing- Pressure [bar]	Cooling Time [s]	Level	Injection Rate [cm^3^/s]	Packing- Pressure [bar]	Cooling Time [s]
1	6.355	6.674	5.687	1	6.526	6.912	5.840	1	6.633	7.017	5.993
2	5.667	5.199	5.513	2	5.815	5.312	5.661	2	5.985	5.479	5.809
3	4.849	4.999	5.672	3	4.989	5.105	5.828	3	5.172	5.293	5.988
Delta	1.506	1.675	0.173	Delta	1.537	1.807	0.179	Delta	1.461	1.724	0.184
Rank	2	1	3	Rank	2	1	3	Rank	2	1	3

**Table 8:** Experiments with constant parameters (lowest cooling time and highest packing pressure) and variable injection rates.

Constant Parameters (B, C)	Injection Rate (A)	Experimental Numbers (#)
20 s–455 bar	56–80–104 cm^3^/s	7, 16, 25

**Table 9:** The values of the bending stress in three experiments and the percentage of increase in structured specimens.

	Bending Stress in Exp. #7 [MPa]	Bending Stress in Exp. #16 [MPa]	Bending Stress in Exp. #25 [MPa]	Differences Due to Process Parameters
Ground structure	34.0	33.5	33.1	2.7%
Perpendicular structure	35.1	34.2	33.8	3.8%
Parallel structure	36.2	35.5	35.3	2.5%
Increase in bending stress (Perpendicular) [%]	3.2%	2.2%	2.2%	
Increase in bending stress (Parallel) [%]	6.5%	6.1%	6.8%	

**Table 10:** The values of the impact bending stress in three experiments and the percentage of increase in structured specimens.

	Impact Strength Exp. #1 [MPa]	Impact Strength Exp. #10 [MPa]	Impact Strength Exp. #19 [MPa]	Differences Due to Process Parameters
Ground surface	7.54	6.79	6.16	22.4%
Perpendicular structure	7.80	7.08	6.34	23.0%
Parallel structure	7.86	7.12	6.50	20.9%
Increase in impact strength (Perpendicular) [%]	3.4%	4.3%	2.9%	
Increase in impact strength (Parallel) [%]	4.2%	4.9%	5.5%	

**Table 11:** Regression analysis—model summaries and the ranks of the significant factors.

	S	R^2^	R^2^ (adj.)	Standardized Effect of Factors
3-point bending				*1. 2. 3*. *(ranks)*
Ground	0.462	96.19%	94.18%	A, B, C
Perpendicular	0.560	95.11%	92.51%	A, B^2^, C
Parallel	0.840	75.61%	62.69%	B, B^2^, C
Impact bending				
Ground	0.202	97.36%	95.97%	B, A, B^2^
Perpendicular	0.202	97.63%	93.82%	B, A, B^2^
Parallel	0.218	97.00%	95.40%	B, A, B^2^

## Data Availability

The data presented in this study are available on request from the corresponding author.
